# *TaKMT-7A* Gene Positively Regulates Spike Number in Wheat

**DOI:** 10.3390/genes17060630

**Published:** 2026-05-30

**Authors:** Qun Wu, Junsheng Sun, Shengfu Yang, Mingxia Zhang, Di Yang, Hao Xue, Haimeng Wu, Ying Guo, Sishen Li, Yanrong An

**Affiliations:** 1 State Key Laboratory of Wheat Improvement, College of Agronomy, Shandong Agricultural University, Tai’an 271018, China; wuqun1213@outlook.com (Q.W.); 15621567685@163.com (J.S.); 19996255553@163.com (S.Y.); zhangmingxia0506@163.com (M.Z.); 18353956757@163.com (D.Y.); 18753815781@163.com (H.X.); 18853855206@163.com (H.W.); guoying729@126.com (Y.G.); 2 Department of Biological and Chemical Engineering, Jining Polytechnic, Jining 272004, China; 3 Jinan Academy of Agricultural Science, Jinan 250316, China

**Keywords:** wheat, spike number per unit area (SN), quantitative trait locus (QTL), histone-lysine N-methyltransferase (KMT), CRISPR/Cas9, overexpression

## Abstract

Wheat (*Triticum aestivum* L.) is a crucial global food crop that plays a central role in agricultural production and food security. The spike number per unit area (SN) is one of the three component factors of grain yield. In this study, we combined the UG-Map with 27 environments of a recombinant inbred line (RIL) population, and mapped a quantitative trait locus (QTL) for SN, *QSn-7A-9048*, in which the meta-QTL interval contains only one candidate gene, *TraesCS7A02G-364700* (*TaKMT-7A*). Using the CRISPR/Cas9 system, we generated two homozygous mutant lines, *aa-1* and *aa-2* of *TaKMT-7A*, which resulted in frameshift mutations, leading to the premature termination of the translation process. The SN values for the wild type (WT), *aa-1*, and *aa-2* were 4.48, 3.43, and 3.48, respectively. Compared with the WT, the SN of the two mutant lines significantly decreased, and no significant differences for grain number per spike (GNS) and thousand-grain weight (TGW) were detected. We also obtained two overexpression (OE) lines of *TaKMT-7A*, OE-1 and OE-2. The SN values for the negative control (NC), OE-1, and OE-2 were 2.31, 3.33, and 3.00, respectively. Compared with NC, the SN values in the OE lines significantly increased. The phenotypes of the knockout (KO) lines and OE lines demonstrate that *TaKMT-7A* acts as a positive regulator of SN in wheat. We performed RNA-Seq analysis using young tiller buds from the WT and *aa-1* mutant lines at the tillering stage, and a total of 2315 differentially expressed genes (DEGs) were identified. We screened 22 wheat genes, of which 18 orthologous genes have previously been cloned and are associated with branching in rice and *Arabidopsis*. These genes included nitrogen transporter, amino metabolism, auxin transporter, auxin homeostasis, auxin response, auxin biosynthesis, strigolactone biosynthesis, and repress gibberellin responses. These genes may represent potential downstream targets of *TaKMT-7A*.

## 1. Introduction

Wheat (*Triticum aestivum* L.) is a crucial global food crop that plays a central role in agricultural production and food security because of its excellent adaptability, outstanding nutritional value, and diverse processing potential [1]. Increasing grain yield is the fundamental objective in wheat breeding programs. The grain yield is directly composed of three factors: the spike number per unit area (SN), grain number per spike (GNS), and the thousand-grain weight (TGW). Tillering, the process of forming lateral shoots from axillary buds, is a fundamental determinant of the SN in wheat. SN stands for the number of effective tillers, the development of which progresses through two independent stages: the initiation of the axillary meristem and the subsequent elongation of the tiller bud.

A great number of QTLs for SN and tiller number (TN) have been mapped [2], such as *QTn.ocs-5A.1* [3], *QTn.ipk-1B* and *QTn.ipk-3B* [4], *QSn.sdau-4B* [5], *QTn.mst-6B* [6], *cqTN-2D.1 and cqTN-4A* [7], *QPtn.sau-4B* [8], and *Qetn-sau-1B.1* [9]. Using the method of map-based cloning and homologous cloning, several SN-related genes were cloned in wheat, including *TaD17* [10], *TaD14* [11], *TaD27* [12], *TaPIL1* [13], *TaSPL14* [14,15,16], *TN1* [17], *LT1* [18], *TaNHLP1* [19], *WT-1* [20], and *Taot2* [21]. These genes collectively form a complex regulatory system involving hormone pathways, transcription cascades, and developmental signals. The number of such genes is limited and still far from sufficient for molecular design breeding. Therefore, other beneficial SN-controlling genes urgently remain to be cloned.

SET (su(var)3-9, enhancer of zeste, trithorax) is a conserved domain across animals and plants. SET-containing proteins typically possess methyltransferase activity, transferring one or multiple methyl groups to the ε-nitrogen of specific lysine residues on histones [22,23,24]. Within the SET family, the SMYD subfamily, which is characterized by a SET domain interrupted by a Zf-MYND (zinc finger MYND, named after Myeloid, Nervy, and DEAF-1), forms a distinct clade with five members in *Arabidopsis thaliana* and six members in *Oryza sativa* [24]. SMYD2, the human homolog of the *Arabidopsis* SMYD proteins ASHR1 and ASHR2, methylates histone H3 at lysine 4 (H_3_K_4_) and lysine 36 (H_3_K_36_), as well as various non-histone proteins, and is essential for the normal development and regulation of multiple pathophysiological processes [25]. Although not yet established in plants, it is likely that plant SMYD2 homologs also possess methyltransferase activity.

In this study, we identified a QTL for SN, *QSn-7A-9048*, using a recombinant inbred line (RIL) population, whose QTL interval covered only one candidate gene, *TraesCS7A02G364700* (*TaKMT-7A*). TaKMT-7A is a typical SMYD subfamily protein whose function was confirmed using CRISPR/Cas9 gene editing and overexpression techniques. The RNA-Seq analyses were carried out to speculate on the regulatory pathways.

## 2. Materials and Methods

### 2.1. Plant Materials and Trial Design

QTL analysis was performed using a set of RILs derived from a cross of ‘Tainong 18 × Linmai 6’ (TN18 × LM6, TL-RILs) [26,27]. The female parent ‘TN18’ is a low-tillering cultivar, developed by our research group in 2008, and became a core parent, with 79 approved cultivars being developed as of 2025. The male parent ‘LM6’ is a high-tillering elite strain developed by the Linyi Academy of Agricultural Sciences, Shandong Province. Field trials (F) were conducted across six growing seasons from 2010 to 2016 (denoted as F(E11)-F(E16)) with two replications [27]. Mineral nutrient trials (M) were carried out in eight nutrient pools during four growing seasons (2012–2016) with two replications. Four treatments were designed: conventional mineral nutrients (CK), low nitrogen (LN), low phosphorus (LP), and low potassium (LK). The environment/treatment combinations were labeled as M(CK13), M(CK14), M(CK15), M(CK16), M(CKAV), etc. [27,28]. All trials were performed at the Experimental Station of Shandong Agricultural University (Tai’an, China). For each replication, the SN was measured the spike number in a 50 cm length within one row and then counting the spike number per m^2^.

The wheat variety Fielder (wild type, WT) was used for CRISPR/Cas9-mediated gene editing. For phenotypic evaluation, the WT, knockout (KO) mutant lines and overexpression (OE) lines were planted in a greenhouse dedicated to transgenic research at the Experimental Station of Shandong Agricultural University. The plants were arranged in rows with a length of 1 m, with 5 cm spacing between plants, 20 seeds per row, and 25 cm spacing between rows. Additionally, tissues from Fielder were collected at different developmental stages for expression pattern analysis.

### 2.2. QTL Analysis

We conducted RNA-Seq analysis for each line of the TL-RIL population. SNPs (single-nucleotide polymorphisms)/InDels were called according to the Chinese Spring RefSeq v1.1 reference genome [29]. Then, the unigene genetic map (UG-Map) was constructed on the basis of the physical positions of unigenes, which can directly obtain the physical position of genes in RefSeq v1.1. The UG-Map comprises 27,452 loci, covering 27,983 unigenes and 118,120 SNP/InDel markers. Among the unigenes, 21,063 were annotated in RefSeq v1.1 (including 16,267 high confidence (HC) genes and 4796 low confidence (LC) genes), 2545 were newly annotated in the TL-RILs (named as *TraesTL1A02G00100*, etc.), and the other 4375 were non-coding RNAs (ncRNAs, named as *STRG_1A.000001*, etc.) [27,28]. QTL analysis was performed using IciMapping 4.1 (https://ics.caas.cn/slyzktz/rj/QTLIciMapping/16954018316940188fb99e52c75f913a.htm), WinQTL Cartographer 2.5 [30] and MapQTL 6.0 [31]. Unigenes located within QTL intervals were designated as candidate genes for the corresponding QTLs.

### 2.3. Gene Editing and Overexpression

We designed a CRISPR-Cas9 target sequence (5′-TCTCTCTCCTCGGCTCCGCAG-GG-3′) for *TaKMT-7A*, which was cloned into the pBUE411 vector for gene editing. To generate *TaKMT-7A* overexpression (OE) wheat lines, the coding DNA sequence (CDS) of *TaKMT-7A* was cloned into the pCAMBIA3301 vector under the control of the *ZmUBIQUITIN* promoter. Both gene editing and overexpression transformations were conducted using Fielder by the Crop Research Institute, Shandong Academy of Agricultural Sciences, China [32]. Homozygous mutant lines of gene editing were selected from T_1_ to T_3_ transgenic lines via high-throughput mutation (Hi-TOM) sequencing [33] for subsequent phenotypic analysis, and transgene expression levels in OE lines were verified by qRT-PCR.

### 2.4. Transient Expression in Nicotiana benthamiana Epidermal Cells

The wheat variety Fielder was used to amplify the full-length cDNA of the target gene. The full-length coding sequence of *TaKMT-7A* was cloned into the recombinant plasmid pCAMBIA1300 using the forward and reverse primers (Forward primer: GAGAACACGGGGGACTCTAGAATGGCCGGCGACACG; reverse primer: CATAAGGGACTGACCACCCGGCTGGTCCATGCTGCATTCAC), to produce TaKMT-7A-GFP fusion protein. The recombinant plasmid p35S::TaKMT-7A-GFP and the control plasmid p35S::GFP were transformed into *Agrobacterium tumefaciens* GV3101. *A. tumefaciens* cells carrying the different constructs were centrifuged and resuspended in an infiltration buffer (10 mM MgCl_2_, 10 mM MES, and 100 μM acetosyringone, pH 5.7) at a final OD600 of 0.4. Samples were infiltrated into 4–5-week-old *N. benthamiana* leaves. GFP was visualized using a laser scanning confocal microscope (Leica TCS SP8; Mannheim, Germany) with an excitation wavelength of 488 nm and a 505–530-nm band-pass emission filter.

### 2.5. RNA Extraction and qRT-PCR Assays

Total RNA was extracted from different tissues of Fielder using the TIANGEN RNA extraction kits (TIANGEN, Beijing, China). First-strand cDNA synthesis was performed with a PrimeScript RT Reagent Kit incorporating genomic DNA removal (Takara Bio, Dalian, China; parent company: Japan). qRT-PCR amplifications were conducted on a CFX96 Real-Time PCR Detection System (Thermo Fisher Scientific, Waltham, MA, USA) employing Taq SYBR Green qPCR Premix (Biosharp Life Sciences, Suzhou, China). The wheat *TaACTIN* gene served as an internal reference, with each assay including three biological replicates and three technical replicates. Gene expression was quantified using the 2^−ΔΔCt^ analytical method [34].

### 2.6. RNA-Seq Analysis

Young tiller buds from the WT and *aa-1* mutant lines at the tillering stage were collected with three biological replications. The RNA libraries were sequenced on the Illumina NovaSeqTM 6000 platform by OE Biotech, Inc., Shanghai, China. Clean reads were aligned to the RefSeq v1.1 reference genome using HISAT2.2.1 [35]. Differentially expressed genes (DEGs) were statistically analyzed using DESeq2_1.52.0 [36], and differential significance was assessed via the negative binomial (NB) distribution test. The thresholds for significantly differential expression were set as q-value < 0.05 and |log2FC| ≥ 1. GO (gene ontology) enrichment analysis and KEGG (Kyoto Encyclopedia of Genes and Genomes) pathway analysis were performed using the OECloud tools (https://cloud.oebiotech.com/task/).

## 3. Results

### 3.1. TaKMT-7A Gene Is a Candidate Gene for SN

**Figure 1:**
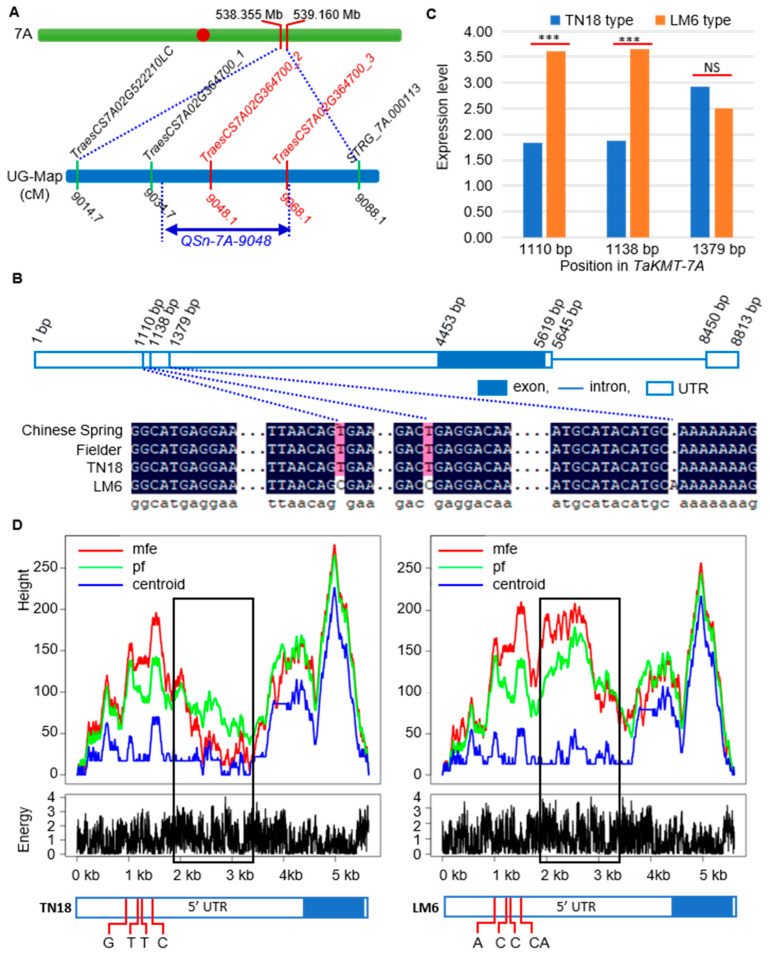
Acquisition of the candidate gene *TaKMT-7A* using QTL mapping. (**A**) QTL interval of *QSn-7A-9048* in the TL-RIL population using three software packages (IciMapping 4.1, WinQTL Cartographer 2.5, and MapQTL 6.0). The candidate gene *TraesCS7A02G364700* (*TaKMT-7A*) is highlighted in red. (**B**) Structure of *TaKMT-7A* gene and its sequence differences between TN18 and LM6. *TaKMT-7A* has three SNPs at 1110 bp, 1138 bp, and 1379 bp, with T/C (TN18/LM6), T/C (TN18/LM6), and C/CA (TN18/LM6) alternates, respectively. Position numbering is performed using the TSS as the +1 bp reference. (**C**) Expression level between the lines of TN18 and LM6 genotypes in TL-RIL population. ***, *p* < 0.001 (Student’s *t*-test). NS, no significant. (**D**) The mountain plot representation of RNA secondary structure prediction for *TaKMT-7A* by RNAFold Webserver (http://rna.tbi.univie.ac.at/cgi-bin/RNAWebSuite/RNAfold.cgi). The black box indicates the region with obvious energy changes. Red line (mfe), minimum free energy structure; green line (pf), generated based on the partition function over all possible RNA secondary structures; blue line, centroid structure or positional entropy, and higher entropy values indicate lower structural stability at that nucleotide position.

Using the UG-Map and 27 environmental data of the TL-RILs [28], a stable QTL for SN, *QSn-7A-9048*, was mapped on chromosome 7A by three QTL analysis software packages, corresponding to a physical region of 538.355–539.160 Mb in the RefSeq v1.1, with an approximate physical span of 0.8 Mb (Figure 1A; Table S1). In WinQTLCart 2.5, the QTL was detected across eight environments, with LOD values ranging from 2.73 to 4.47, and phenotypic variance explained (PVE) ranging from 9.27% to 22.16%. In IciMapping 4.1, the QTL was detected in two environments, with LOD values of 2.84 and 3.00, and PVE values of 2.97% and 7.94%, respectively. In MapQTL 6.0, the QTL was detected in five environments, with LOD values ranging from 2.63 to 3.78 and PVE ranging from 6.40% to 9.30%. The additive effect of *QSn-7A-9048* was negative, indicating that the allele increasing SN came from the male parent, LM6, of the TL-RIL population.

The meta-QTL interval was 9037.5–9069.5 cM in the UG-Map, which covered only one candidate gene, *TraesCS7A02G364700* (*TraesCS7A02G364700-2* and *TraesCS7A02G 364700-3*) (Figure 1A). This gene is annotated as a histone-lysine N-methyltransferase and is named *TaKMT-7A*. *TraesCS7A02G364700-2* has two SNPs at 1110 bp and 1138 bp, both with T/C (TN18/LM6) alternates, and *TraesCS7A02G364700-3* has one InDel at 1379 bp with a C/CA (TN18/LM6) alternate. These three sites are all caused by 5’UTR mutants (Figure 1B). The expression level of *TraesCS7A02G364700-2* was significantly different between the lines of the TN18 and LM6 types in the TL-RIL population, and that of *TraesCS7A-02G364700-3* was not significantly different (Figure 1C). RNA secondary structure prediction analysis indicated that *TaKMT-7A^AA^* in TN18 has a significantly lower minimum free energy (MFE) in the 1.9kb to 2.3kb region compared to *TaKMT-7A^BB^* in LM6, reflecting a more stable topological structure (Figure 1D).

### 3.2. Functional Validation of TaKMT-7A Using Gene Editing and Overexpression

**Figure 2:**
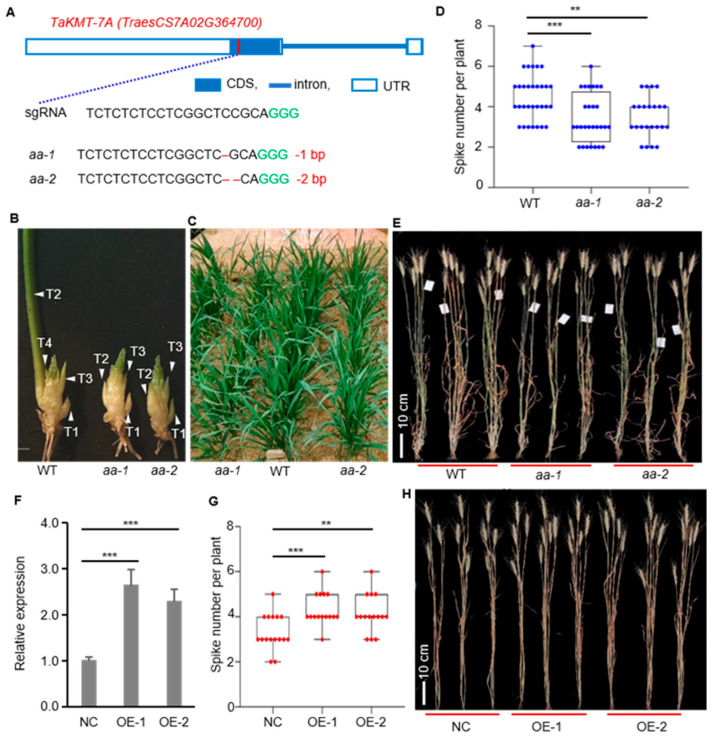
*TaKMT-7A* functional confirmation using CRISPR/Cas9 and overexpression technologies. (**A**) The gene-editing sgRNA is at the CDS 5′ end; mutants *aa-1* and *aa-2* carry a 1-bp and 2-bp deletion within the sgRNA, respectively. (**B**) Tiller buds following leaf removal at the tillering stage. White triangles indicate the position of each tiller bud. T1, tiller bud from the axil of the first leaf; T2, from the axil of the second leaf; etc. (**C**) Less tillering of mutant plants compared to WT at the tillering stage. (**D**) Boxplots of SN for WT and mutant lines. (**E**) Whole-plant comparison of mature WT and mutant individuals. (**F**) Comparison of *TaKMT-7A* expression levels in NC and OE lines. (**G**) Boxplots of SN for NC and OE lines. (**H**) Whole-plant comparison of mature NC and OE individuals. WT, wild type; NC, negative control, representing null segregants derived from the transgenic line; OE, *TaKMT-7A* overexpression line. **, *p* < 0.01; ***, *p* < 0.001 (Student’s *t*-test).

To validate the function of *TaKMT-7A* in SN regulation, we performed gene editing using the CRISPR/Cas9 system in the wheat variety Fielder. Sequence mutations in the mutant lines were identified by Hi-TOM analysis. In the T_3_ generation, we acquired two homozygous knockout (KO) mutant lines, *aa-1* (-1 bp) and *aa-2* (-2 bp) (Figure 2A). The *aa-1* mutant has a single-base C deletion at CDS position 389, and *aa-2* has a two-base CG deletion at positions 389–390. Both mutant lines exhibited frameshift mutations, leading to premature termination of the translation process (Figure S1).

In terms of phenotype, the development of the mutant lines *aa-1* and *aa-2* was delayed compared with that of the WT (Figure 2B), and the tiller number (TN) was obviously different at the tillering stage (Figure 2C). We measured the yield traits of the two mutant lines. The SN values for WT, *aa-1*, and *aa-2* were 4.48, 3.43, and 3.48, respectively. Compared with the WT, the two mutant lines presented significant decreases of 1.05 and 1.00 (Figure 2D,E). No significant differences for GNS and TGW were detected.

We further generated *TaKMT-7A* overexpression lines OE-1 and OE-2, and their expression levels were measured by qRT-PCR. The expression levels of the negative control (NC), OE-1, and OE-2 were 1.0, 2.5, and 2.4, respectively (Figure 2F), with significant differences between the NC and OE lines. The SN values for NC, OE-1, and OE-2 were 2.31, 3.33, and 3.00, respectively. Compared with NC, the OE lines exhibited significantly increased SN values of 1.02 and 0.69 (Figure 2G,H).

The phenotypes of the KO lines and OE lines demonstrate that *TaKMT-7A* acts as a positive regulator of the SN in wheat.

### 3.3. Characterization of the TaKMT-7A Gene

**Figure 3:**
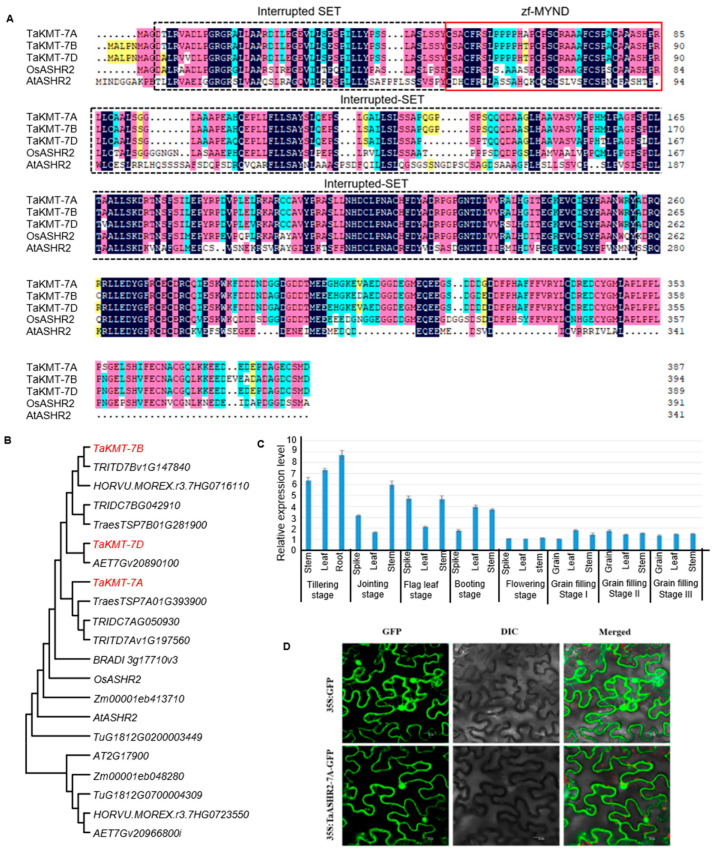
Domain, evolution, expression, and subcellular localization of the *TaKMT-7A* gene. (**A**) TaKMT has an N-terminal SET domain (the dashed box) interrupted by a zf-MYND domain (the solid red box), as well as its orhologs. OsASHR2 and AtASHR2, orthologs of TaKMT in rice and Arabidopsis, respectively. (**B**) Phylogenetic tree of *TaKMT-7A* (red) and its homologous genes across diverse plant species. These genes all encode SYMD proteins. (**C**) Expression pattern of *TaKMT-7A* gene across different tissues and developmental stages in wheat variety Fielder. Values are means (*n* = 3). (**D**) Subcellular localization of TaKMT-7A-GFP in *N. benthamiana* leaf epidermal cells. The TaKMT-7A-GFP fusion protein localized to the plasma membrane, cytoplasm, and nucleus, similar to the GFP alone control.

The full length of gDNA for the *TaKMT-7A* gene is 8813 bp, and the full length of cDNA is 6008 bp. The *TaKMT-7A* gene contains one exon of 1167 bp and one intron of 2805 bp, with a 5′-UTR of 4452 bp and a 3′-UTR of 389 bp, encoding 388 amino acids. *TaKMT-7A* possesses an unusually long 5′ UTR and intron, suggesting that *TaKMT-7A* is subject to more extensive transcriptional or translational regulation. Similar to its homologs in *Arabidopsis* and rice, *TaKMT-7A* possesses a characteristic SMYD architecture: an N-terminal SET domain interrupted by a putative 36-amino-acid zf-MYND domain (Figure 3A).

Orthologous genes of *TaKMT-7A* were retrieved using the Ensembl Plants database, and a phylogenetic tree was constructed. The results showed that *TaKMT-7A* and its homologous genes, *TaKMT-7B* (*TraesCS7B02G262900*) and *TaKMT-7D* (*TraesCS7D02G358100*), are classified in distinct clades. *TaKMT-7A* is clustered most closely with *TraesTSP7A01G393900*, *TRIDC7AG050930*, and *TRITD7Av1G197560*; *TaKMT-7B* with *TRITD7Bv1G147840* and *HORVU.MOREX.r3.7HG0716110*; and *TaKMT-7D* with *AET7Gv20890100* (Figure 3B). Cross-species collinearity analysis was performed on the 7A chromosomal region harboring the *TaKMT-7A* gene. The region was highly conserved in *Triticeae* species and in the B and D subgenomes of common wheat, whereas an inversion event was detected in the A subgenome (Figure S2).

qRT-PCR analysis revealed that *TaKMT-7A* is a constitutively expressed gene with an expression peak in root tissue at the tillering stage. The expression levels were relatively high in the stem tissue at the tillering and jointing stages, which are the critical periods for spike number development (Figure 3C). This spatiotemporal expression pattern provides physiological support for the role of *TaKMT-7A* in regulating SN. We performed subcellular localization of the *TaKMT-7A* protein using a transient expression system in tobacco (*N. benthamiana*) epidermal cells. The results showed that *TaKMT-7A* is broadly distributed in most cellular compartments except the vacuole (Figure 3D), suggesting that *TaKMT-7A* has a wide range of substrate types, likely targeting both nuclear histones and cytoplasmic non-histone proteins.

### 3.4. Transcriptomic Analysis of Differentially Expressed Genes

To explore the molecular pathways through which the *TaKMT-7A* gene regulates SN, we performed RNA-Seq analysis using young tiller buds from the WT and *aa-1* mutant lines at the tillering stage. A total of 2315 differentially expressed genes (DEGs) were identified. Among these genes, 1502 were upregulated, and 813 were downregulated (Figure S3).

GO analysis revealed that the upregulated DEGs were significantly enriched in biological processes such as nucleosome assembly, DNA replication initiation, DNA repair, cell cycle, DNA recombination, and DNA methylation on cytosine within a CG sequence. The downregulated DEGs were enriched in fatty acid biosynthetic processes, pollen tube development, response to light stimulus, and response to cold (Figure S4). KEGG analysis revealed that the upregulated DEGs were significantly enriched in pathways such as DNA replication, homologous recombination, mismatch repair, base excision repair, and pyrimidine metabolism. The downregulated DEGs were enriched in fatty acid elongation, biosynthesis of unsaturated fatty acids, phenylpropanoid biosynthesis, cutin, suberin, and wax biosynthesis, as well as a plant–pathogen interaction (Figure S5).

**Figure 4:**
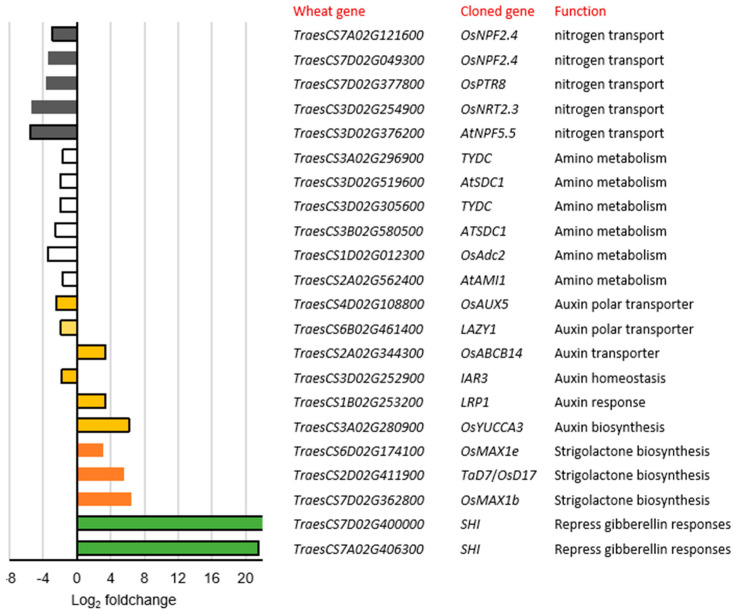
Orthologous genes previously cloned that are associated with branching in rice and *Arabidopsis*. The references for the genes listed in the figure are as follows: *OsNPF2.4* [37], *OsPTR8* [38], *OsNRT2.3* [39], *AtNPF5.5* [40], *TYDC* [41], *AtSDC1* [42], *OsAdc2* [43], *AtAMI1* [44], *OsAUX5* [45], *LAZY1* [46], *IAR3* [47], *OsABCB14* [47], *LAR3* [48], *LRP1* [49], *OsYUCCA3* [45], *OsMAX1e* [50], *OsCCD7* [10,50], *OsMAX1b* [50] and SHI [51].

Importantly, from the 2315 DEGs, we screened 22 wheat genes, of which 18 orthologous genes have previously been cloned and associated with branching in rice and *Arabidopsis* (Figure 4). Among them, the downregulated DEGs included nitrogen transporter (*OsNPF2.4*, *OsPTR8*, *OsNRT2.3*, and *AtNPF5.5*), amino metabolism (*TYDC*, *AtSDC1, OsAdc2*, and *AtAMI1*), auxin polar transporter (*OsAUX5* and *LAZY1*), and auxin homeostasis (*IAR3*). The upregulated DEGs included auxin transporter (*OsABCB14*), auxin homeostasis (*LAR3*), auxin response (*LRP1*), auxin biosynthesis (*OsYUCCA3*), strigolactone biosynthesis (*OsMAX1e*, *OsCCD7*, and *OsMAX1b*), and repress gibberellin responses (*SHI*) (Figure 4). These orthologs represent potential downstream targets of *TaKMT-7A*.

## 4. Discussion

Wheat is an allohexaploid species and has a very large and highly repetitive genome. We previously constructed a UG-Map of the TL-RIL population with 27,983 genes on the basis of the physical positions of genes from the RefSeq v1.1 reference genome [28]. Combined with 27 environments, we mapped a QTL for SN, *QSn-7A-9048*, in which the meta-QTL interval contains only one candidate gene, *TaKMT-7A*. We subsequently confirmed that *TaKMT-7A* regulated SN using gene editing and overexpression. We previously cloned and validated several genes from the QTLs based on the UG-Map of the TL-RIL population, such as *TaXIP* [52], *TaDHL* [53], and *PI4KA* [54]. This approach should be an effective method for cloning genes from wheat.

In this study, we successfully identified and validated that *TaKMT-7A* acts as a positive regulator of SN in wheat, which has not been reported previously in wheat and other plants. TaKMT-7A is a member of the SMYD subfamily of SET domain-containing proteins and can methylate H_3_K_4_ and/or H_3_K_36_, as well as a few non-histone proteins [55]. This indicates that tillering is regulated by dynamic histone and non-histone methylation modifications, which are highly consistent with previous epigenomic studies in wheat and rice. Strigolactones act as essential upstream inhibitors of tiller development and trigger the central regulatory factor *TB1*, a key suppressor of axillary bud growth [56,57]. It has been demonstrated that strigolactone signaling genes are modified through histone methylation by the transcription factor NGR5 responding to endogenous nitrogen [58]. Similarly, glucose promotes axillary bud outgrowth by activating the TOR kinase pathway through establishing H_3_K_27_me_3_ modification on bud-suppressive genes, including *RAX1*, *RSB1*, *HB21*, *BRC1* (*TB1* homolog), and *TIE1* [59]. Epigenetic regulation is important in mediating cultivar-specific adaptation to LN in wheat. Low nitrogen (LN) induces H3K27ac and H3K27me3 in tiller-related genes, such as *DWARF27* (*TaD27*), *PIN-LIKES 7* (*TaPILS7*), and *LONELY GUY 4* (*TaLOG4*), significantly in KN9204 [60]. Overall, plants orchestrate genome-wide epigenetic modulation of tillering-related genes in response to endogenous and exogenous signals. In this study, differential expression analysis between the mutant and WT indicates that *TaKMT-7A* affects the expression of several tillering-related genes. The nuclear and cytoplasmic localization of TaKMT-7A implies its potential involvement in the methylation of both nuclear histones and cytoplasmic proteins. Thus, dissecting whether and how TaKMT-7A epigenetically modulates the expression of tillering-related genes will shed light on its regulatory mechanism in tillering.

From 2315 DEGs identified by RNA-Seq analysis, we selected 22 wheat genes, of which 18 orthologous genes have previously been cloned and associated with branching in both rice and *Arabidopsis*. These genes may represent potential downstream targets of *TaKMT-7A*. More importantly, as long-range signals, strigolactones act as central inhibitors to suppress excessive tillering, mediate rice developmental responses to phosphate and nitrate deficiency, and modulate auxin transport from shoots to roots [56,61,62]. In rice, the strigolactone precursor is synthesized by D27 (a carotenoid isomerase) together with the carotenoid cleavage enzymes D10/OsCCD8 and D17/HTD1/OsCCD7 [56,63]. In wheat, *TaD17*, *TaD27*, and *TaD14* negatively regulate tillering through strigolactone synthesis and reception [10,11,12]. In this study, among the 18 orthologous genes previously cloned, *D17*, *MAX1b*, and *OsMAX1e* are involved in strigolactones biosynthesis [50,64], *LAZY1*, *OsAUX5*, and *ABCB14* are involved in auxin transport and distribution [45,46,47], while *PTR8*, *NRT2.3*, and *NPF2.4* participate in nitrogen absorption and transport processes [37,38,39]. In contrast, other well-characterized wheat tillering regulatory genes were not detected in the DEG dataset. These excluded genes include *TN1* [17], *WT-1*, *LT1* [18,20], *TIN7*, and *Taot2* [19,21]. Therefore, the strigolactone biosynthesis, auxin signal, nitrogen absorption, and transport might be the passways through which *TaKMT-7A* modulates wheat spike number. Further experimental validation is required to obtain direct molecular evidence.

## Data Availability

The original contributions presented in the study are included in the article/Supplementary Materials. Further inquiries can be directed to the corresponding authors.

## Supplementary Materials

The following supporting information can be downloaded at: https://www.mdpi.com/article/10.3390/genes17060630/s1, Figure S1: Amino acid sequence alignment after *TaKMT-7A* gene editing; Figure S2: Collinearity analysis of *TaKMT-7A* gene (marked by the red arrow) across thirteen species; Figure S3: Volcano map of differentially expressed genes in tillering node of *TaKMT-7A* mutant line *aa-1* and WT; Figure S4: Top 30 enriched GO terms; Figure S5: Top 20 enriched KEGG pathways (bubble plot). Table S1: QTL results of three software programs for *QSn-7A-9048*.

## References

[1] Yao Y., Guo W., Gou J., Hu Z., Liu J., Ma J., Zong Y., Xin M., Chen W., Li Q. (2025). Wheat 2035: Integrating pan-omics and advanced biotechnology for future wheat design. Mol. Plant.

[2] Cao S., Xu D., Hanif M., Xia X., He Z. (2020). Genetic architecture underpinning yield component traits in wheat. Theor. Appl. Genet..

[3] Kato K., Miura H., Sawada S. (2000). Mapping QTLs controlling grain yield and its components on chromosome 5A of wheat. Theor. Appl. Genet..

[4] Huang X., Cöster H., Ganal M.W., Röder M.S. (2003). Advanced backcross QTL analysis for the identification of quantitative trait loci alleles from wild relatives of wheat (*Triticum aestivum* L.). Theor. Appl. Genet..

[5] Deng S., Wu X., Wu Y., Zhou R., Wang H., Jia J., Liu S. (2011). Characterization and precise mapping of a QTL increasing spike number with pleiotropic effects in wheat. Theor. Appl. Genet..

[6] Naruoka Y., Talbert L.E., Lanning S.P., Blake N.K., Martin J.M., Sherman J.D. (2011). Identification of quantitative trait loci for productive tiller number and its relationship to agronomic traits in spring wheat. Theor. Appl. Genet..

[7] Ren T., Hu Y., Tang Y., Li C., Yan B., Ren Z., Tan F., Tang Z., Fu S., Li Z. (2018). Utilization of a Wheat55K SNP array for mapping of major QTL for tiller number. Front. Plant Sci..

[8] Liu J., Luo W., Qin N., Ding P., Zhang H., Yang C., Mu Y., Tang H., Liu Y., Li W. (2018). A 55 K SNP array-based genetic map and its utilization in QTL mapping for productive tiller number in common wheat. Theor. Appl. Genet..

[9] Liu J., Tang H., Qu X., Liu H., Li C., Tu Y., Li S., Habib A., Mu Y., Dai S. (2020). A novel, major, and validated QTL for the effective tiller number located on chromosome arm 1BL in bread wheat. Plant Mol. Biol..

[10] Sigalas P.P., Bennett T., Buchner P., Thomas S.G., Jamois F., Arkoun M., Yvin J.C., Bennett M.J., Hawkesford M.J. (2024). At the crossroads: Strigolactones mediate changes in cytokinin synthesis and signalling in response to nitrogen limitation. Plant J..

[11] Liu R., Hou J., Li H., Xu P., Zhang Z., Zhang X. (2021). Association of TaD14-4D, a gene involved in strigolactone signaling, with yield contributing traits in wheat. Int. J. Mol. Sci..

[12] Awan M.J.A., Amin I., Rasheed A., Saeed N.A., Mansoor S. (2024). Knockout mutation in TaD27 enhances number of productive tillers in hexaploid wheat. Front. Genome Ed..

[13] Zhang L., He G., Li Y., Yang Z., Liu T., Xie X., Kong X., Sun J. (2022). PIL transcription factors directly interact with SPLs and repress tillering/branching in plants. New Phytol..

[14] Jian C., Pan Y., Liu S., Guo M., Huang Y., Cao L., Zhang W., Yan L., Zhang X., Hou J. (2024). The TaGW2-TaSPL14 module regulates the trade-off between tiller number and grain weight in wheat. J. Integr. Plant Biol..

[15] Cao J., Liu K., Song W., Zhang J., Yao Y., Xin M., Hu Z., Peng H., Ni Z., Sun Q. (2021). Pleiotropic function of the SQUAMOSA PROMOTER-BINDING PROTEIN-LIKE gene TaSPL14 in wheat plant architecture. Planta.

[16] Cao L., Li T., Geng S., Zhang Y., Pan Y., Zhang X., Wang F., Hao C. (2023). TaSPL14-7A is a conserved regulator controlling plant architecture and yield traits in common wheat (*Triticum aestivum* L.). Front. Plant Sci..

[17] Dong C., Zhang L., Zhang Q., Yang Y., Li D., Xie Z., Cui G., Chen Y., Wu L., Li Z. (2023). Tiller number 1 encodes an ankyrin repeat protein that controls tillering in bread wheat. Nat. Commun..

[18] Yuan Y., Lyu B., Qi J., Liu X., Wang Y., Delaplace P., Du Y. (2024). A novel regulator of wheat tilleringLT1identified by using an upgraded BSA method, uni-BSA. Mol. Breed..

[19] Si Y., Tian S., Niu J., Lu Q., Shang Q., Ma S., Zhang Z., Du T., Wu H., Li J. (2025). The TaNHLP1-TaRACK1A module regulates tillering via abscisic acid signaling in wheat. Nat. Commun..

[20] Maqbool R., Nagarajan R., Mutti J.S., Gill K.S. (2025). Wheat Tiller -1 (WT-1), a GRAS domain encoding gene, controls both tillering and spikelet number in wheat. Plant Mol. Biol..

[21] Wang C., Bai J., Xiong H., Xie Y., Gu J., Zhao L., Li H., Ding Y., Guo X., Guo H. (2025). Characterization of wheat oligo-tiller mutant ot2 and fine mapping of the mutant gene Taot2. Plant Genome.

[22] Zhang Y., Reinberg D. (2001). Transcription regulation by histone methylation: Interplay between different covalent modifications of the core histone tails. Genes Dev..

[23] Lachner M., Jenuwein T. (2002). The many faces of histone lysine methylation. Curr. Opin. Cell Biol..

[24] Zhou H., Liu Y., Liang Y., Zhou D., Li S., Lin S., Dong H., Huang L. (2020). The function of histone lysine methylation related SET domain group proteins in plants. Protein Sci..

[25] Yi X., Jiang X.J., Fang Z.M. (2019). Histone methyltransferase SMYD2: Ubiquitous regulator of disease. Clin. Epigenet..

[26] Zhang M. (2019). Construction of Genetic Map for Unigenes and QTL Mapping for Nitrogen Use Efficiency in Wheat. Doctoral Thesis.

[27] Zhang M., Han X., Wang H., Sun J., Guo B., Gao M., Xu H., Zhang G., Li H., Cao X. (2024). Efficient cloning of genes for wheat yield component traits from QTLs via sequencing of the RIL population. bioRxiv.

[28] Han X., Zhang M., Gao M., Yuan Y., Yuan Y., Zhang G., An Y., Guo Y., Kong F., Li S. (2023). QTL mapping and candidate gene identifying for N, P, and K use efficiency at the maturity stages in wheat. Genes.

[29] IWGSC (2018). Shifting the limits in wheat research and breeding using a fully annotated reference genome. Science.

[30] Wang S., Basten C.J., Zeng Z. (2006). Windows QTL Cartographer 2.5.

[31] Van Ooijen J. (2009). MapQTL 6: Software for the mapping of quantitative trait loci in experimental populations. Software Manual.

[32] Zhang S., Zhang R., Gao J., Song G., Li J., Li W., Qi Y., Li Y., Li G. (2021). CRISPR/Cas9-mediated genome editing for wheat grain quality improvement. Plant Biotechnol. J..

[33] Liu Q., Wang C., Jiao X., Zhang H., Song L., Li Y., Gao C., Wang K. (2019). Hi-TOM: A platform for high-throughput tracking of mutations induced by CRISPR/Cas systems. Sci. China Life Sci..

[34] Livak K., Schmittgen T. (2001). Analysis of relative gene expression data using real-time quantitative PCR and the 2^−ΔΔCT^ method. Methods.

[35] Kim D., Langmead B., Salzberg S. (2015). HISAT: A fast spliced aligner with low memory requirements. Nat. Methods.

[36] Love M., Huber W., Anders S. (2014). Moderated estimation of fold change and dispersion for RNA-seq data with DESeq2. Genome Biol..

[37] Xia X., Fan X., Wei J., Feng H., Qu H., Xie D., Miller A.J., Xu G. (2015). Rice nitrate transporter OsNPF2.4 functions in low-affinity acquisition and long-distance transport. J. Exp. Bot..

[38] Rentsch D., Laloi M., Rouhara I., Schmelzer E., Delrot S., Frommer W.B. (1995). NTR1 encodes a high affinity oligopeptide transporter in Arabidopsis. FEBS Lett..

[39] Tang Z., Fan X., Li Q., Feng H., Miller A.J., Shen Q., Xu G. (2012). Knockdown of a rice stelar nitrate transporter alters long-distance translocation but not root influx. Plant Physiol..

[40] Léran S., Garg B., Boursiac Y., Corratgé-Faillie C., Brachet C., Tillard P., Gojon A., Lacombe B. (2015). AtNPF5.5, a nitrate transporter affecting nitrogen accumulation in Arabidopsis embryo. Sci. Rep..

[41] Kang S., Kang K., Lee K., Back K. (2007). Characterization of rice tryptophan decarboxylases and their direct involvement in serotonin biosynthesis in transgenic rice. Planta.

[42] Kwon Y., Yu S.I., Lee H., Yim J.H., Zhu J.K., Lee B.H. (2012). Arabidopsis serine decarboxylase mutants implicate the roles of ethanolamine in plant growth and development. Int. J. Mol. Sci..

[43] Sánchez-Rangel D., Chávez-Martínez A.I., Rodríguez-Hernández A.A., Maruri-López I., Urano K., Shinozaki K., Jiménez-Bremont J.F. (2016). Simultaneous silencing of two arginine decarboxylase genes alters development in Arabidopsis. Front. Plant Sci..

[44] Gao Y., Dai X., Aoi Y., Takebayashi Y., Yang L., Guo X., Zeng Q., Yu H., Kasahara H., Zhao Y. (2020). Two homologous INDOLE-3-ACETAMIDE (IAM) HYDROLASE genes are required for the auxin effects of IAM in Arabidopsis. J. Genet. Genom..

[45] Huang X., Lu Z., Zhai L., Li N., Yan H. (2023). The small auxin-up RNA SAUR10 is involved in the promotion of seedling growth in rice. Plants.

[46] Zhang N., Yu H., Yu H., Cai Y., Huang L., Xu C., Xiong G., Meng X., Wang J., Chen H. (2018). A core regulatory pathway controlling rice tiller angle mediated by the LAZY1-dependent asymmetric distribution of auxin. Plant Cell.

[47] Xu Y., Zhang S., Guo H., Wang S., Xu L., Li C., Qian Q., Chen F., Geisler M., Qi Y. (2014). OsABCB14 functions in auxin transport and iron homeostasis in rice (*Oryza sativa* L.). Plant J..

[48] Davies R.T., Goetz D.H., Lasswell J., Anderson M.N., Bartel B. (1999). IAR3 encodes an auxin conjugate hydrolase from Arabidopsis. Plant Cell.

[49] Ullah H., Chen J.G., Temple B., Boyes D.C., Alonso J.M., Davis K.R., Ecker J.R., Jones A.M. (2003). The beta-subunit of the Arabidopsis G protein negatively regulates auxin-induced cell division and affects multiple developmental processes. Plant Cell.

[50] Cui J., Nishide N., Mashiguchi K., Kuroha K., Miya M., Sugimoto K., Itoh J.I., Yamaguchi S., Izawa T. (2023). Fertilization controls tiller numbers via transcriptional regulation of a MAX1-like gene in rice cultivation. Nat. Commun..

[51] Fridborg I., Kuusk S., Moritz T., Sundberg E. (1999). The Arabidopsis dwarf mutantshi exhibits reduced gibberellin responses conferred by overexpression of a new putative zinc finger protein. Plant Cell.

[52] Sun Z., Zhang M., An Y., Han X., Guo B., Lv G., Zhao Y., Guo Y., Li S. (2022). CRISPR/Cas9-mediated disruption of xylanase inhibitor protein (XIP) gene improved the dough quality of common wheat. Front. Plant Sci..

[53] Guo B., Jin X., Chen J., Xu H., Zhang M., Lu X., Wu R., Zhao Y., Guo Y., An Y. (2022). ATP-dependent DNA helicase (TaDHL), a novel reduced-height (Rht) gene in wheat. Genes.

[54] Zhang H., Ma L., Zhai Y., Wang H., Niu M., Zhang M., Guo Y., An Y., Li S., Zhao Y. (2025). A phosphatidylinositol 4-kinase alpha (PI4KA) gene reduces plant height of common wheat. Gene.

[55] Padilla A., Manganaro J.F., Huesgen L., Roess D.A., Brown M.A., Crans D.C. (2023). Targeting epigenetic changes mediated by members of the SMYD family of lysine methyltransferases. Molecules.

[56] Zhou F., Lin Q., Zhu L., Ren Y., Zhou K., Shabek N., Wu F., Mao H., Dong W., Gan L. (2013). D14-SCF(D3)-dependent degradation of D53 regulates strigolactonesignalling. Nature.

[57] Choi M.S., Woo M.O., Koh E.B., Lee J., Ham T.H., Seo H.S., Koh H.J. (2012). Teosinte Branched 1 modulates tillering in rice plants. Plant Cell Rep..

[58] Wu K., Wang S., Song W., Zhang J., Wang Y., Liu Q., Yu J., Ye Y., Li S., Chen J. (2020). Enhanced sustainable green revolution yield via nitrogen-responsive chromatin modulation in rice. Science.

[59] Ye R., Wang M., Du H., Chhajed S., Koh J., Liu K.H., Shin J., Wu Y., Shi L., Xu L. (2022). Glucose-driven TOR–FIE–PRC2 signalling controls plant development. Nature.

[60] Zhang H., Jin Z., Cui F., Zhao L., Zhang X., Chen J., Zhang J., Li Y., Li Y., Niu Y. (2023). Epigenetic modifications regulate cultivar-specific root development and metabolic adaptation to nitrogen availability in wheat. Nat. Commun..

[61] Jiang D., Hao X., Zhang J., Tang H., Wang F. (2021). Reducing expression of TaOTUB1s decreases tiller number in wheat. Plant Signal. Behav..

[62] Sun H., Tao J., Liu S., Huang S., Chen S., Xie X., Yoneyama K., Zhang Y., Xu G. (2014). Strigolactones are involved in phosphate- and nitrate-deficiency-induced root development and auxin transport in rice. J. Exp. Bot..

[63] Lin H., Wang R., Qian Q., Yan M., Meng X., Fu Z., Yan C., Jiang B., Su Z., Li J. (2009). DWARF27, an iron-containing protein required for the biosynthesis of strigolactones, regulates rice tiller bud outgrowth. Plant Cell.

[64] Huang X., Kuang Z., Zhou R., Liu T., Tang L., Gao Z., Liu T., Fan X., Xuan W., Luo L. (2025). Mutation of strigolactone biosynthetic gene DWARF 17 impairs the responses of rice tillering to N supply. Plant J..

