# Supplementary material for: TaKMT-7A Gene Positively Regulates Spike Number in Wheat
Source: Genes (Basel). 2026 May 30;17(6):630. doi: 10.3390/genes17060630 (PMC13300514; doi:10.3390/genes17060630)
Supplement: Supplementary file 1 [file genes-17-00630-s001.zip › Table S1.pdf]

Table S1 QTL results of three software programs for *QSn-7A-9048*

Trait	Environment	Peak position	Left position	Right position	Meta-left	Meta-right	LOD	R^2(%)	Additive effect
Windows QTL cartographer 2.5									
SN	F(AV)	9059.1	9050.1	9074.1	9048.2	9052.2	4.16	16.42	(46.31)
SN	F(E15)	9059.1	9051.1	9065.6			4.47	22.16	(107.40)
SN	F(E16)	9053.6	9043.7	9066.1			3.10	11.70	(49.86)
SN	M(LK16)	9054.6	9042.2	9065.6			2.65	11.19	(58.64)
SN	M(LKAV)	9054.6	9043.2	9063.6			3.75	14.84	(39.14)
SN	M(LN14)	9063.1	9053.1	9074.6			2.76	13.70	(40.88)
SN	M(LN16)	9054.6	9040.7	9063.1			3.82	14.95	(82.90)
SN	M(LNAV)	9060.6	9048.1	9075.1			3.48	13.31	(39.34)
SN	M(LP16)	9051.1	9040.2	9062.1			2.73	9.27	(56.74)
SN	M(LPAV)	9046.2	9038.2	9054.6			3.59	12.47	(30.93)
IciMapping 4.1									
SN	M(LN14)	9062.3	9052.1	9073.4	9041.8	9069.5	2.84	2.97	(35.23)
SN	M(LNAV)	9063.8	9052.7	9068.1			3.00	7.94	(35.72)
MapQTL 6.0									
SN	F(AV)	9059.1	9049.1	9068.1	9037.5	9061.3	3.78	9.30	(53.98)
SN	F(E15)	9057.1	9042.7	9068.1			2.72	6.60	(88.88)
SN	F(E16)	9054.1	9042.7	9066.1			2.63	6.40	(53.21)
SN	M(LN16)	9056.1	9041.7	9066.1			3.11	7.50	(88.87)
SN	M(LNAV)	9062.1	9053.1	9076.1			3.59	9.00	(49.58)
