# Supplementary figures and images for: TaKMT-7A Gene Positively Regulates Spike Number in Wheat
Source: Genes (Basel). 2026 May 30;17(6):630. doi: 10.3390/genes17060630 (PMC13300514; doi:10.3390/genes17060630)

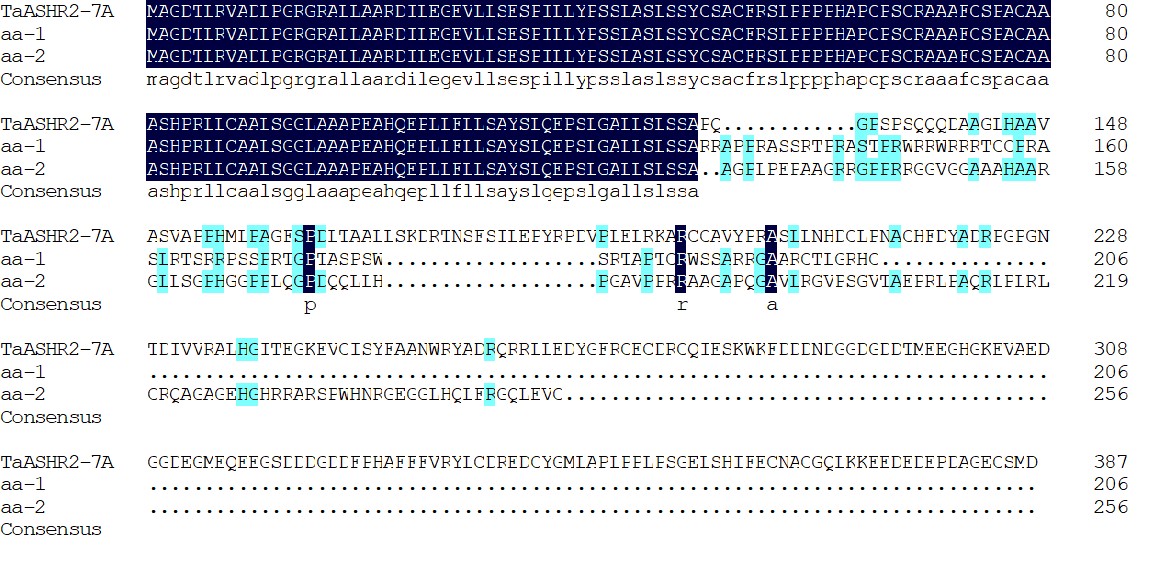

Supplement: Supplementary file 1 [file genes-17-00630-s001.zip › Figure S1.jpg]

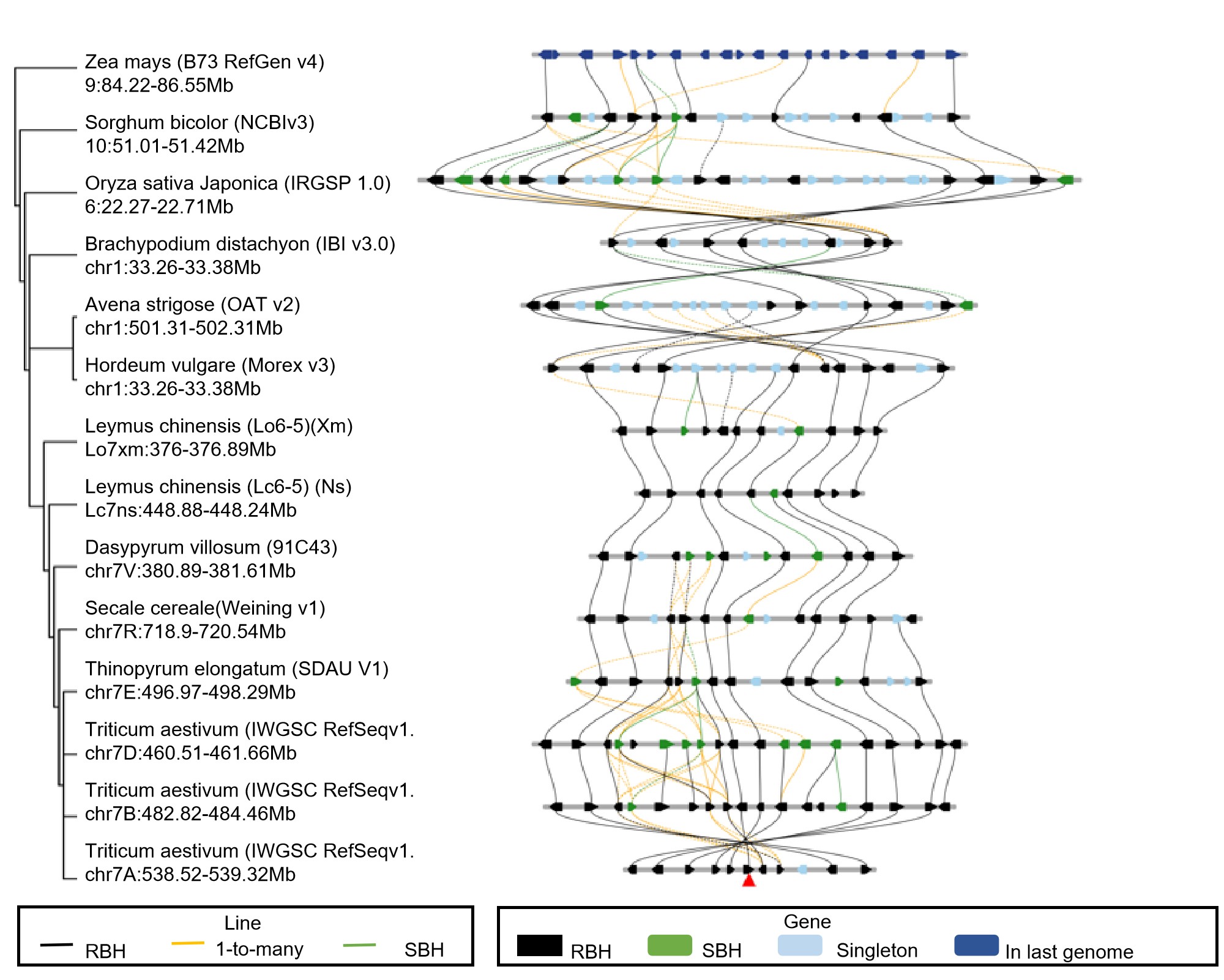

Supplement: Supplementary file 1 [file genes-17-00630-s001.zip › Figure S2.jpg]

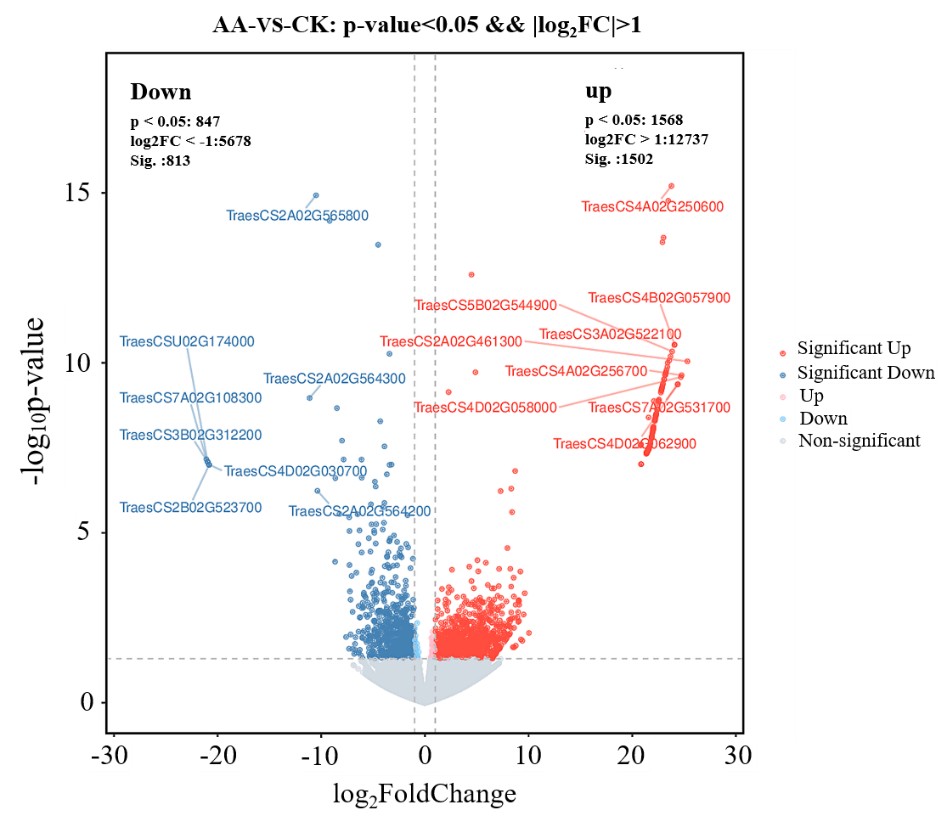

Supplement: Supplementary file 1 [file genes-17-00630-s001.zip › Figure S3.jpg]

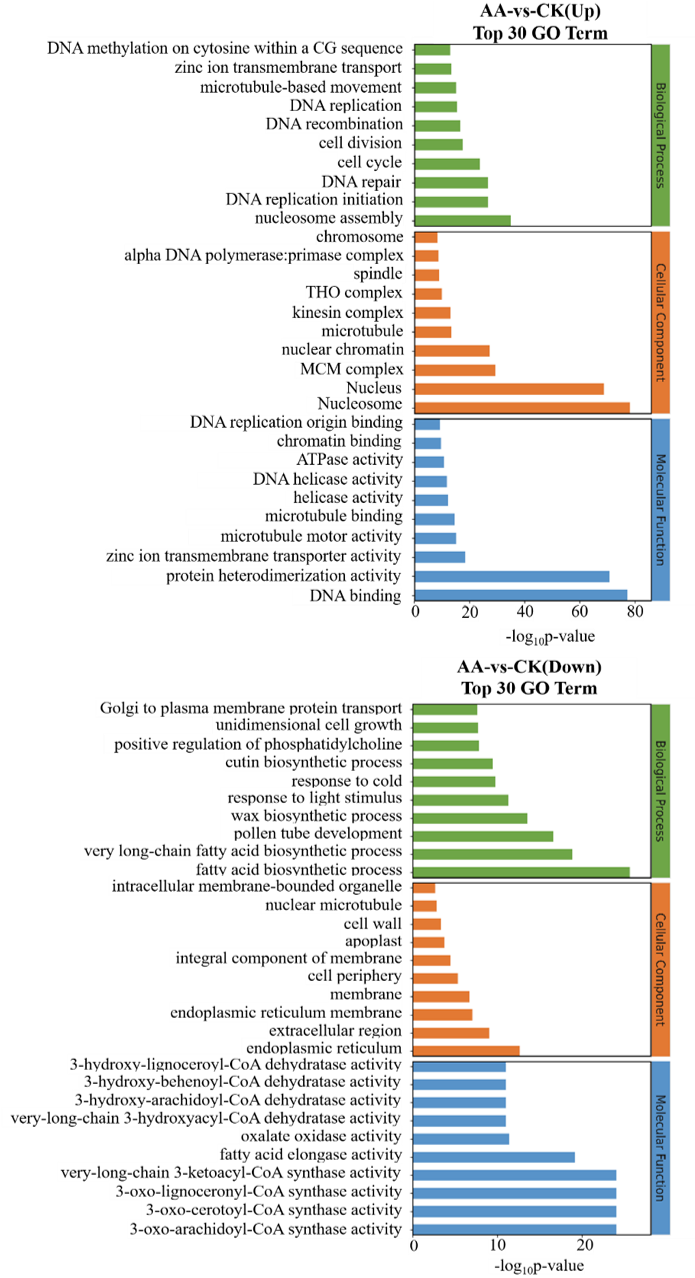

Supplement: Supplementary file 1 [file genes-17-00630-s001.zip › Figure S4.png]

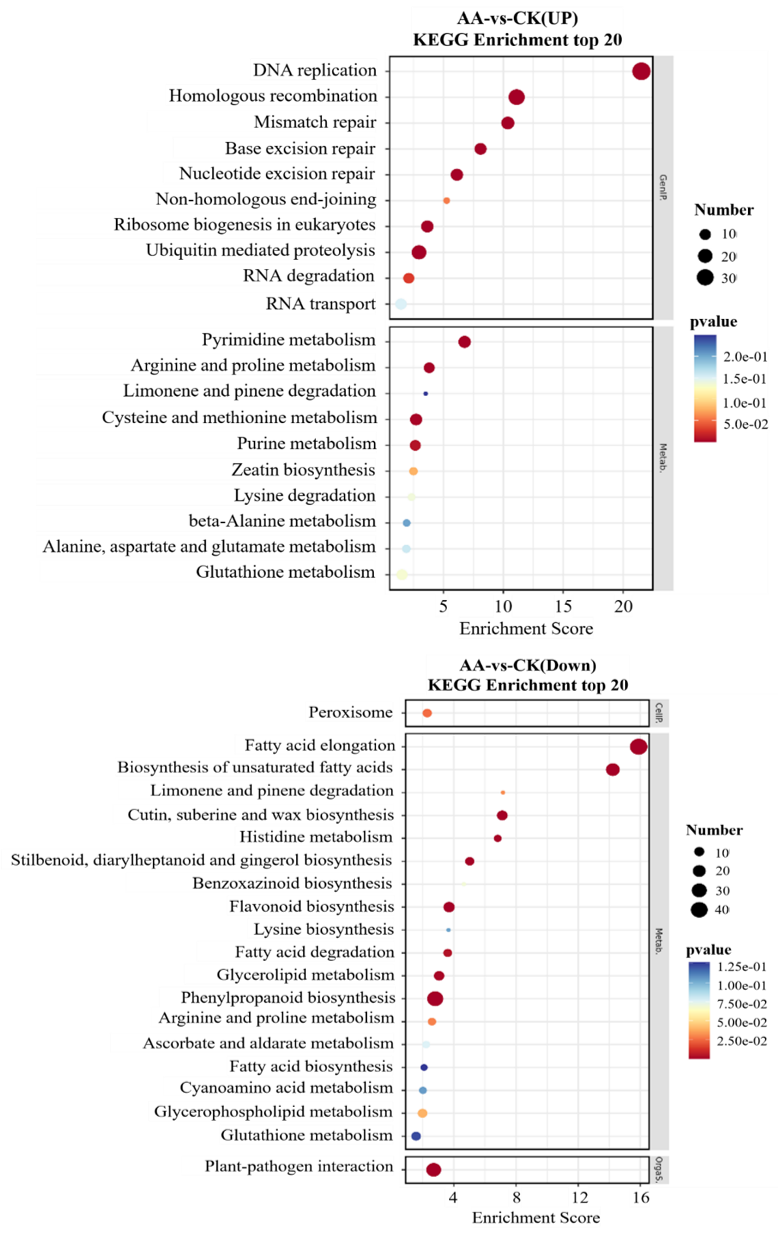

Supplement: Supplementary file 1 [file genes-17-00630-s001.zip › Figure S5.png]
